# Antigen expression on recurrent meningioma cells

**DOI:** 10.2478/v10019-010-0028-6

**Published:** 2010-05-24

**Authors:** Andrej Vranic

**Affiliations:** Department of Neurosurgery, University Medical Centre Ljubljana, Ljubljana, Slovenia

**Keywords:** meningioma, recurrence, tumour markers, proliferation index, MIB-1 antigen, cathepsin B, cathepsin L

## Abstract

**Introduction:**

Meningiomas are intracranial brain tumours that frequently recur. Recurrence rates up to 20% in 20 years for benign meningiomas, up to 80% for atypical meningiomas and up to 100% for malignant meningiomas, have been reported. The most important prognostic factors for meningioma recurrence are meningioma grade, meningioma invasiveness and radicality of neurosurgical resection. The aim of our study was to evaluate the differences in antigenic expression on the surface of meningioma cells between recurrent and non-recurrent meningiomas.

**Methods:**

19 recurrent meningiomas and 35 non-recurrent meningiomas were compared regarding the expression of MIB-1 antigen, progesterone receptors, cathepsin B and cathepsin L, using immunohistochemistry.

**Results:**

MIB-1 antigen expression was higher in the recurrent meningioma group (p=0.001). No difference in progesterone receptor status between recurrent and non-recurrent meningiomas was confirmed. Immunohistochemical intensity scores for cathepsin B (p= 0.007) and cathepsin L (p<0.001) were both higher in the recurrent than in the non-recurrent meningioma group.

**Conslusions:**

MIB-1 antigen expression is higher in recurrent compared to non-recurrent meningiomas. There is no difference in expression of progesterone receptors between recurrent and non-recurrent meningiomas. Cathepsins B and L are expressed more in recurrent meningiomas.

## Introduction

Meningiomas represent 10–20% of primary intracranial tumours and show a wide range of histomorphological subtypes. According to signs of malignancy in their histological picture, they are classified by the WHO classification as benign meningiomas (BM - grade I), atypical meningiomas (AM - grade II) and malignant meningiomas (MM - grade III). Although in general meningiomas are considered as slow-growing benign tumours, recurrence rates are quite high. Over a 20 year period, the recurrence rate for BM is reported to be 10–26%, for AM 50–80% and for MM 78–100%.^1^–^4^ The average recurrence-free interval ranges between 4 and 6.5 years. The most important risk factors for the meningioma recurrence are the meningioma grade, meningioma invasiveness and thoroughness of neurosurgical resection, which is a principal approach for any surgical treatment in the brain tumour patient.^5^,^6^ Meningioma invasiveness has been observed in all meningioma grades.^1^

Biological markers with the prognostic value for the meningioma recurrence have been sought in the last decades. The proliferation index Ki67 (Ki67 index), assessed as the percentage of MIB-1 positive cells in the area of greatest proliferation, is the most frequently used proliferation index for meningiomas. The Ki67 index was shown to correlate with higher malignancy grades of meningiomas and was observed to be significantly more expressed in recurrent meningiomas compared to non-recurrent ones.^7^ However, the Ki67 index has not been confirmed as a statistically significant predictor of recurrence in gross-totally removed benign meningiomas.^8^,^9^

The loss of progesterone receptors (PR), which are normally expressed in two thirds of BM, seems to correlate with a higher meningioma grade and the decreased recurrence-free survival time of patients with meningiomas.^9^–^12^ Other biochemical factors, like the epithelial membrane antigen, S100, activated caspase 3 and its inhibitor survivin, HER2, metalloproteases MMP-2 and MMP-9 and others have all been proposed as tumour markers significant for the meningioma recurrence.^13^–^19^

Cathepsins are intracellular cysteine proteases normally present in most tissues. A high expression of cathepsins B and L was found in several malignant tumours.^20^–^24^ A high expression of cathepsin D was observed in benign meningiomas.^25^ A higher expression of cathepsins B in L was found in AM and MM2 3,27, compared to BM. Cathepsins B and L were found to be expressed more in recurrent meningiomas.^26^,^28^ However, only three recurrent meningiomas were studied and the need for the evaluation of cathepsins B and L expression on a larger series of recurrent meningiomas, was emphasized.^28^

In the present study, we focused on expression of the MIB-1 antigen, PR, cathepsin B and cathepsin L on meningioma cells. The aim of the study was to compare recurrent and non-recurrent meningiomas. Differences between three meningioma grades were also sought.

## Patients and methods

In our retrospective study, 54 patients (32 female and 22 male), aged from 19 to 78 (mean 55.5 years), operated for meningioma at the University Medical Centre Ljubljana in the years 1996 – 2001, were selected randomly for the study. Thirty-three meningiomas were diagnosed as BM, 11 as AM and 10 as MM. Meningiomas were operated on by different surgeons and they were described by surgeons as gross-totally removed, graded as Simpson I or Simpson II. All meningiomas were intracranial: 24 cranial base, 16 hemispheric and 14 parasagittal meningiomas were studied. Nineteen patients were in the recurrent meningioma group, and 35 patients were in the non-recurrent meningioma group. In the recurrent meningioma group, the mean time to the recurrence from the first operation was 4.5 years (1–14 years). In the non-recurrent meningioma group, tumours have not recurred for at least 5–10 years (mean 6.5 years).

Tissue samples were fixed in formaldehyde and embedded in paraffin wax. 5μm thick sections stained with haematoxylin and eosin (H&E) were assessed by the pathologist with regard to grade of malignancy according to the WHO classification. In the recurrent meningioma group, 6 tumours (34%) were benign, 5 (23%) were atypical and 8 (43%) were malignant. In the non-recurrent group, 27 (75%) were benign, 6 (18%) were atypical and 2 (7%) were malignant.

Immunohistochemistry (IH) was performed on tissue sections cut from the most representative paraffin block of each tumour using the standard procedures. IH was performed according to the routine protocols used in everyday practice at the Institute of Pathology, Ljubljana. Primary mouse monoclonal antibodies against human cathepsin B and human cathepsin L (Krka, Novo mesto), primary mouse anti-human Ki67 monoclonal antibodies (clone MIB-1, No 7240, DAKO, Denmark) and primary mouse anti-human progesterone receptor antibodies (clone PgR 636, No M3569 diluted, DAKO, Denmark) were used. Antibodies were incubated with slides for 26 min at 40°C. The Ki67 index was calculated with the help of the Leica Q Prodit computer program (Leica, Germany), counting the percentage of MIB-1 labelled nuclei in the most affected region. Positive or negative progesterone receptor status was determined. Tumours were considered positive even if there were only a few cells with a positive IH reaction. The intensity of the IH reaction between cathepsin B and L antibodies and the tumour cells was scored from 0 to 5 by two independent observers. Cases with a different score at the beginning were discussed and the agreement was reached. Intensity and frequency of immunostaining for cathepsins B and L was scored with: 0 no staining; 1 very mild; 2 mild, 3 moderate, 4 strong and 5 very strong staining observed.

Statistical analysis was performed, using SPSS 16.0 for Windows (SPSS Inc., USA). The recurrent meningioma group was compared with the non-recurrent one. Variables used in the analysis included meningioma grade, absence or presence of recurrence, proliferation index, PR status, and IH intensity scores for cathepsins B and L. Differences in expression of biological markers were analyzed using independent-samples T-test. Significance of differences between recurrent and non-recurrent meningiomas, as well as between different histological subgroups, was given as the p value; p<0.05 was considered significant.

## Results

### Differences between recurrent and non-recurrent meningiomas

#### Proliferation index

The proliferation index was higher in the recurrent meningioma group (p=0.001), regardless of the tumour grade.

#### Progesterone receptor status

No differences in PR status between recurrent and non-recurrent meningiomas were confirmed. Ten out of 19 recurrent and 18 out of 35 non-recurrent meningiomas had a positive PR status. PR status did not correlate with other biological markers.

#### Protein expression of cathepsins B and L

IH intensity scores for cathepsin B (p=0.007) and cathepsin L (p<0.001) were both higher in the recurrent than in the non-recurrent meningioma group.

### Differences between meningioma grades

**FIGURE 1:**
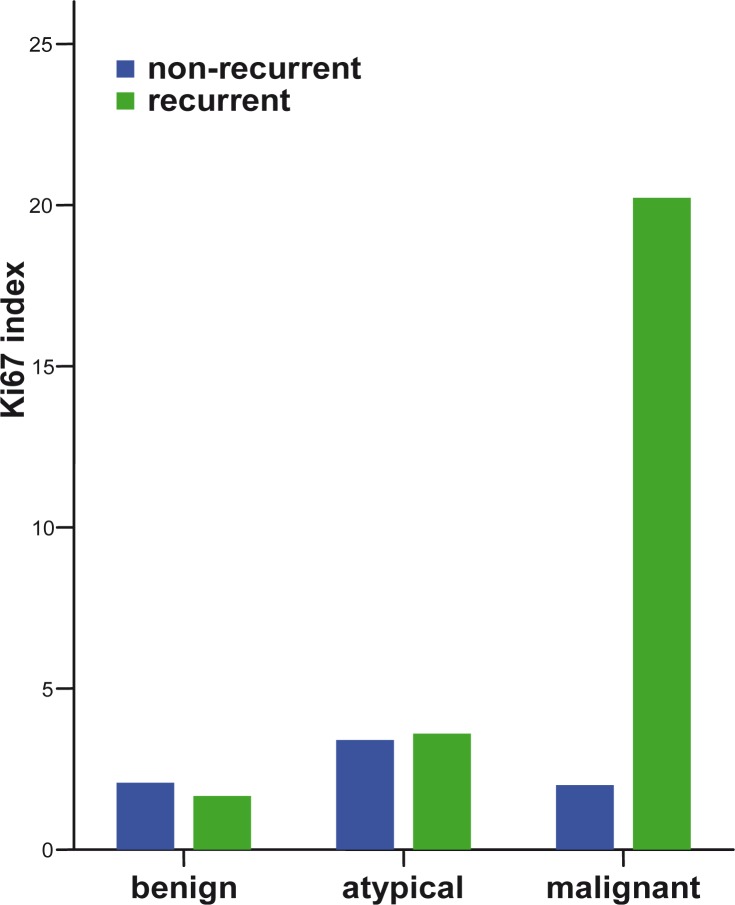
Expression of MIB-1 antigen in different meningioma grades comparing non-recurrent and recurrent meningiomas.

**FIGURE 2:**
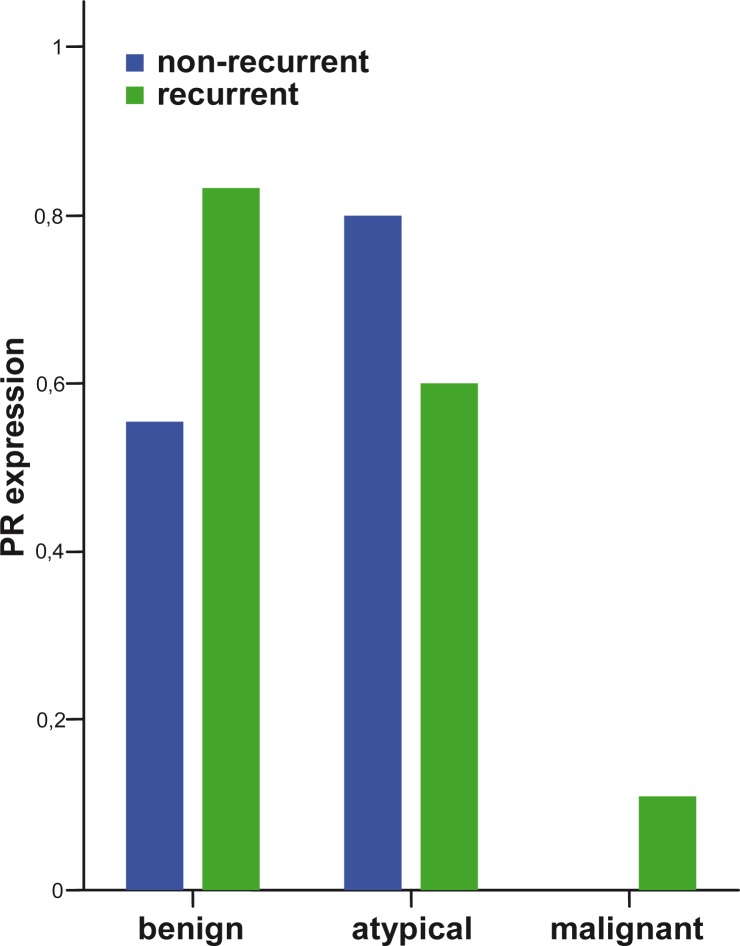
Expression of PR in different meningioma grades comparing non-recurrent and recurrent meningiomas.

**FIGURE 3:**
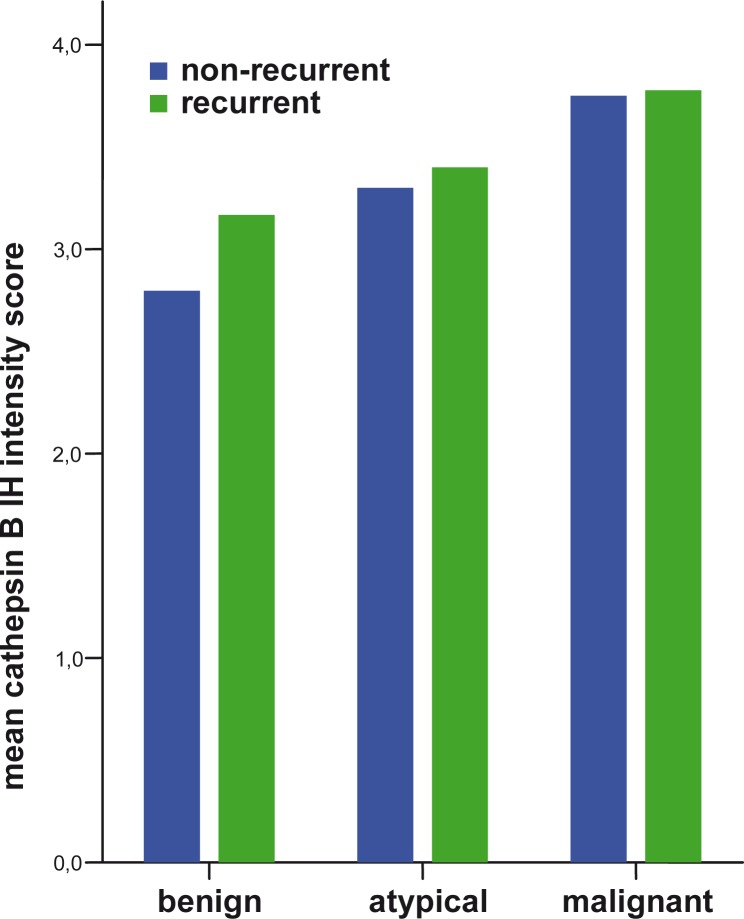
IH intensity score of cathepsin B expression in different meningioma grades comparing non-recurrent and recurrent meningiomas.

**FIGURE 4:**
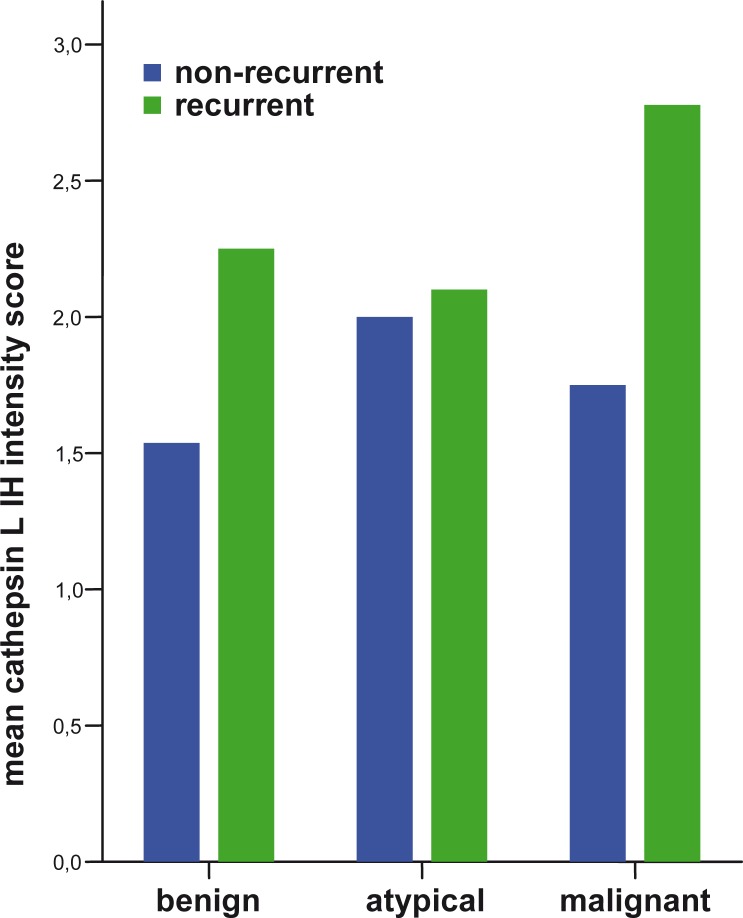
IH intensity score of cathepsin L expression in different meningioma grades comparing non-recurrent and recurrent meningiomas.

The proliferation index increased with the meningioma grade (Figure 1). A higher proliferation index (p=0.006) and a significant loss of PR (p=0.002) were observed in MM compared to BM (Figure 2). Higher IH intensity scores for cathepsin B (p=0.007), and for cathepsin L (p=0.006), were observed in MM compared to BM (Figures 3 and 4).

### Differences between recurrent and non-recurrent meningiomas in the BM subgroup

In the subgroup of 33 BM, recurrent BM expressed more cathepsin L (p=0.035) than non-recurrent BM.

## Discussion

In our series of 54 meningiomas, treated in a single institution, we showed that MIB-1 antigen, cathepsin B and cathepsin L were expressed more in recurrent compared to non-recurrent meningiomas. No difference in PR expression between recurrent and non-recurrent meningiomas was noticed.

Comparing different meningioma grades, we showed that higher meningioma grades express more MIB-1 antigen, less PR and more cathepsins B and L. Differences between BM and MM were statistically important in all four parameters. Differences between BM and AM as well as differences between AM and MM were statistically insignificant.

Although often studied and even used in everyday diagnostics of meningiomas, the prognostic significance of the Ki67 index remains poorly defined.^9^,^29^,^30^ The Ki67 index significantly increases from BM through AM to MM, but there is a considerable overlap between different grades.^9^,^29^,^31^ In a series of primary meningiomas of all three grades, Perry *et al.* reported a Ki67 index ≥ 4.2% associated with the decreased recurrence-free survival in univariate but not in multivariate analysis.^29^

No correlation between the outcome and the Ki-67 index was found in 600 cases of benign meningiomas.^9^ The principal limitation of the Ki67 index seems to be the lack of standardization of the technique and difficulties in defining cut-off values.^9^,^29^,^30^ Our study confirms an important role of the Ki67 index in meningioma grading, suggesting different cell proliferation rates in different meningioma grades (Figure 1).

No differences in PR expression between recurrent and non-recurrent meningiomas were found in our study. PR were expressed more frequently in female patients` meningiomas. A significant loss of PR was observed in MM (Figure 2). The loss of PR could be responsible for malignant progression of meningiomas with PR acting protectively. According to our observations, meningioma recurrences are most frequent for female patients at the onset of menopause. More research work is required to confirm this observation.

Suggesting the invasive nature of recurrent meningiomas, most research work has been focused on proteases. Lysosomal proteases cathepsin B and L have been associated with tumour invasiveness.^20^–^22^ They were considered as factors contributing to invasiveness of meningiomas.^26^–^28^ Higher expressions of cathepsin B, metalloprotease-2 and metalloprotease-9 were also detected in meningiomas, histologically described as invasive.^18^,^32^

In the present study, we found that cathepsins B and L were expressed more in recurrent meningiomas compared to non-recurrent ones. This finding is in accordance with previous reports about cathepsin expression in recurrent meningiomas which so far have not been confirmed on a larger series.^28^ Our study shows obvious differences between the two groups, suggesting recurrent meningiomas were biologically different – *i.e.* more invasive than non-recurrent meningiomas. Our findings support the idea of cathepsins as indicators of invasiveness of meningiomas. They suggest that meningiomas recur not only due to a higher cell proliferation marked by the Ki67 index but also due to a more powerful invasiveness of meningioma cells, marked by higher cathepsin B and L expression.

Our study also confirms that higher expressions of cathepsins B and L correlate with a higher meningioma grade, as already shown in previous studies.^26^–^28^ However, since invasiveness is also found in BM, the meningioma grade is probably not directly correlated to expression of cathepsins B and L. Several cellular mechanisms of cellular proliferation, invasiveness and others are responsible for the malignant transformation of meningiomas.

The expression of antigenic markers,the Ki67 index and cathepsins B and L seem to correlate with a tendency of meningioma to recur. Measuring the expression of these three antigens on meningioma cells could have prognostic value already at the first appearance of a meningioma. These antigenic markers could be proposed as prognostic indicators of the recurrence-free survival of patients with meningiomas. A larger multivariate study on a larger population is needed to confirm the prognostic value of MIB-1 antigen, PR, cathepsin B and cathepsin L on meningioma cells.

A higher probability of an individual meningioma to recur would mean an alarm to start a more aggressive therapeutic approach for the patient. This would include more frequent control checkups, more frequent control MRI scans and possibly immediate postoperative irradiation with the proper radiation delivery.^33^ A more frequent follow-up is particularly important since the median time to recurrence in meningiomas is rather long (4.5 years), which gives us enough time for therapeutic intervention.

## Conclusions

The recurrence rate of meningiomas, especially of AM and MM is quite high. So far, the most important known prognostic factors for the meningioma recurrence have been meningioma grade, meningioma invasiveness and completeness of neurosurgical resection. Since the WHO grading system alone does not always correctly predict the biological behaviour of meningiomas, antigenic markers with prognostic significance are being sought. The MIB-1 antigen, cathepsin B and cathepsin L are shown to be expressed more on cells of recurrent meningiomas compared to non-recurrent ones. Expression of these antigens could possibly help us to assess the risk of meningioma recurrence with each individual meningioma patient.
